# Identifying and Addressing the Underlying Core Problems in Healthcare Environments: An Illustration Using an Emergency Department Game

**DOI:** 10.3390/ijerph181910083

**Published:** 2021-09-25

**Authors:** Gustavo M. Bacelar-Silva, James F. Cox, Humberto R. Baptista, Pedro Pereira Rodrigues

**Affiliations:** 1 Department of Community Medicine, Information and Health Decision Sciences, Faculty of Medicine (MEDCIDS-FMUP), University of Porto, 4200-450 Porto, Portugal; pprodrigues@med.up.pt; 2 Center for Health Technology and Services Research (CINTESIS), 4200-450 Porto, Portugal; 3 Department of Distance Learning, Bahiana School of Medicine and Public Health, Salvador 40285-001, Brazil; 4 Management Department, Terry College of Business, University of Georgia, Athens, GA 30602, USA; jcox@uga.edu; 5 Vectis Solutions, São Paulo 04088-004, Brazil; humberto@vectis-solutions.com

**Keywords:** hospital emergency service, hospital administration, delivery of health care, problem-based learning, board game, theory of constraints

## Abstract

The emergency department (ED) crowding is a critical healthcare issue worldwide that leads to long waits and poorer healthcare outcomes. Goldratt’s theory of constraints (TOC) has been used effectively to improve such problematic environments for more than three decades. While most TOC solutions are simple, with many viewing them as purely common sense, they represent paradigm shifts in how to manage complex, uncertain, and silo environments. Goldratt used a simple dice game with a straight flow (I-shape) to illustrate the impact of dependent resources and statistical fluctuations in managing resources. Additionally, games help to overcome resistance to change and gain ownership by having participants develop their solutions. This new cooperative game illustrates an ED environment where patients may follow different care pathways according to their clinical needs, timeliness of care is measured in minutes, the demand is highly uncertain, and treatment must frequently start almost immediately. A Monte Carlo simulation validated the TOC solution to this ED game, achieving results similar to the real TOC’s implementations. Moreover, this article provides a thorough process to Socratically introduce TOC to healthcare professionals and others to recognize that the EDs’ (like other healthcare systems’) core problem is the traditional approach to managing them.

## 1. Introduction

An emergency department (ED) is a medical facility where emergency patients can receive timely and specialized care without prior appointments, but it is not the reality in many healthcare organizations. ED crowding is a critical healthcare issue worldwide, and it has been worsening over time. Crowding in the ED leads to long waits, which means longer lengths of stay, delayed care, poor treatment outcomes, and even death. Several studies have reported adverse consequences of ED crowding for both patients and staff [1,2,3,4].

ED crowding affects patients in many ways. It increases delay in medical assessment and adequate treatment, which may prolong physical and emotional suffering, contribute to worsening clinical conditions and avoidable complications, elevate exposure to error, and, ultimately, increase mortality [1,2,3,4]. Staff experience increased stress levels, including exposure to violence, and reduced adherence to clinical guidelines during ED crowding [2]. On the system level, crowding increases the length of stay beyond the ED. It increases the length of stay of admitted patients and their treatment costs while reducing hospital revenue [2,5].

Few studies have reported effective solutions to ED crowding. Friedman and Pauly [6] correctly stated that EDs must have some protective capacity to support statistical fluctuations. Nevertheless, like almost all the proposed solutions, their solution involves paying more for more resources. The general belief is that the crowding problem is a consequence of a shortage of resources (e.g., beds, medical providers) [7,8].

This belief in the “lack of capacity due to a shortage of resources” is not an exclusive problem of EDs. It is present in many healthcare environments, such as primary care appointments [9,10], hospital admissions and discharges [11], and elective surgeries [12]. However, before paying for more resources, an underlying assumption needs to be checked: are currently available resources utilized as effectively as possible? One management approach distinguishes itself by attaining protective capacity by better managing the existing resources: the theory of constraints (TOC) [13,14].

### 1.1. The Theory of Constraints and Its Application in Health Care

Outcomes of the TOC’s implementations in healthcare services, including in the ED, have already demonstrated that these organizations usually have a huge amount of wasted protective capacity. As an example, after implementing the TOC at the Oxfordshire Radcliffe Hospital [15,16], the percentage of patients spending less than 4 h in the Accident and Emergency department increased from an average that varied between 50% and 60% to over 95% in 2 months—with no extra staff—in a period where demand increased by 40% due to government changes decreasing availability of GPs to acute cases. TOC experts provide similar results, like Knight [17,18], Stratton and Knight [19], and Sierraalta [20], who successfully demonstrated how to significantly improve EDs using the existing resources by applying the TOC.

The TOC is a holistic management approach. Goldratt, the father of TOC, considered any human organization to be a system composed of dependent processes which have different capacities and are subject to statistical fluctuations. In this context, the process with the least capacity determines the system’s throughput; it is the system’s constraint [21]. For that reason, the TOC’s improvement focus is on the system’s constraint. This is the leverage point for managing (planning, scheduling, executing, controlling, measuring, and improving) the system so that other parts operate in a synchronized way to support the constraint (global optimum) instead of as individual parts trying to achieve their own goals (local optima) [22].

The TOC has three processes of ongoing improvement (POOGI) to achieve high utilization of the constraint and seamless flow of patients through the system [22]. This study used two of them: the Five Focusing Steps (5FS) and Buffer Management. We explain and illustrate the use of these two POOGIs further within the article.

### 1.2. A Game to Support the TOC in Health Care

Although most TOC solutions are based on common sense, they represent paradigm shifts in how to manage complex, uncertain, and silo systems. Unfortunately, the TOC is still not the common practice to manage healthcare resources and patient flows. As an effort to Socratically introduce the basic concepts and demonstrate how dependent processes and statistical fluctuations interact with patients flows in health care, Knight and West [23] used Goldratt’s dice game and introduced an online dice game simulator at the 2014 TOCICO Annual Conference. However, Goldratt’s dice game has an I-shape—an environment where materials flow in a linear sequence of processes [24]. Such a shape does not illustrate an ED nor most healthcare environments where patients may have different treatment plans (requiring specific resources) according to their clinical needs. Hence, healthcare professionals sometimes comment, “We’re unique, that won’t work here.”

This new ED game represents one out of many different healthcare environments that can illustrate the facts of life that exist in any organization subject to dependent events and statistical fluctuations. Most, if not all, healthcare workers and most of the public are familiar with the ED environment through personal experience or the dramatized TV programs that center around ED operations. Alternatively, the game could be around the increasing backlogs in elective surgery, the outpatient appointment scheduling problem, or the growing patient no-show rates, for instance.

The ED Game illustrates some of the characteristics that make managing an ED unique: uncertain and highly variable demand of resources; the criticality of addressing patients’ needs in minutes or a few hours (versus weeks and months in other environments); the number of different pathways a patient may follow and the decision concerning which pathway is required as the patient is treated; and prioritizing and sequencing of the tasks.

The purpose of this research was to introduce the ED Game and provide a guide to academics, practitioners, and consultants who want to:

1. Demonstrate the impact of statistical fluctuations and dependent processes in a complex healthcare environment;
2. Demonstrate how bad/outdated (traditional) management policies block patient treatment and flow;
3. Support the introduction of the basic TOC concepts and tools to healthcare audiences;
4. Nurture participants (players) to improve patient flows in the ED and other healthcare environments using the TOC.

The Material and Methods section provides an overview of the game, some of its rules, and the statistical analyses of various scenarios. The Results section describes the experience of running this game as part of a workshop held at the end of 2019 at a Faculty of Medicine—the audience consisted of physicians, C-level managers, a consultant, a pharmacist, and IT professionals. After describing the gameplay of a shift, we provide the simulation results that validate the statistical analysis and the description of the debriefing session, where the facilitator introduces the TOC concepts and Socratically stimulates participants to analyze the system and develop solutions to play the next shift. The Discussion section provides an interpretation of how this game can support achieving successful TOC’s implementations to address the chronic problems of healthcare services. The Conclusion section summarizes the research and its contributions.

## 2. Materials and Methods

**Figure 1:**
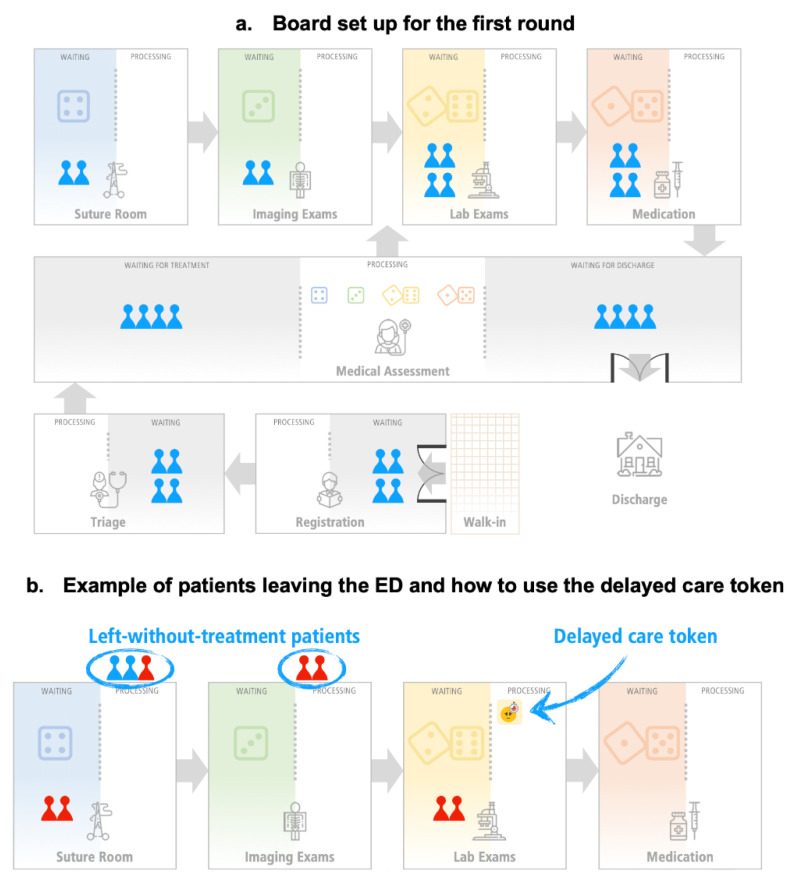
(**a**) Example of how to initially set up the board to play. (**b**) Implications of the patient-“batching” policy; notice some left-without-treatment patients (LWOT) on top of SUTURE ROOM and IMAGING EXAMS, while LAB EXAMS is at risk of receiving its second delayed care token.

In this ED game, the players must deliver care to emergency patients (with specific treatment plans) by directing their flow accordingly through seven resources till the end of the ED episode, where patients go to the DISCHARGE area. See Figure 1a for the layout of the ED. Each resource has a waiting area for patients awaiting processing at that resource. The general flow starts upon walking into the ED (WALK-IN), the patient is processed at REGISTRATION and follows to TRIAGE and MEDICAL ASSESSMENT. Once MEDICAL ASSESSMENT processes patients for the first time (those waiting in the grey area on the left side), they follow through different treatment plans (paths) starting from (a) SUTURE ROOM, (b) IMAGING EXAMS, (c) LAB EXAMS, and (d) MEDICATION. After being processed at the appropriate specialized resource(s), the patient goes back to MEDICAL ASSESSMENT before DISCHARGE. MEDICAL ASSESSMENT must both treat and discharge patients.

The game illustrates the destination after the ED as DISCHARGE for two reasons. First, to reduce the complexities of managing other potential bottlenecks (operating theaters, beds, staffing in specialized wards, ICUs, etc.) throughout a hospital complex, which might cause backups and crowding of the ED, and potential diversion of patients to other hospitals. The second reason is that home is the most frequent destination after an ED visit, almost 90% of the time in the United States in 2017 [25].

The game is played in three shifts, with 10 rounds per shift. Each round, each player rolls a die to determine the number of patients in their area that they can process/treat that round. Each round lasts 48 min; 10 rounds represent an 8 h shift. Including the discussion and learning, the game takes approximately 1.5 h to play. Figure 1a shows the ED board with patients awaiting processing at the various resources prepared for the first round. Detailed rules are available in the Supplementary Materials File S1.

In the first shift, the participants are free to play according to their prior knowledge. Next, the facilitator introduces the Five Focusing Steps (5FS) and Buffer Management. The facilitator and the participants discuss and apply the first three steps (how to identify and exploit the constraint and adjust the system accordingly) in the second shift. Before the third shift, the participants discuss step 4 (elevate the constraint) and apply it in the sequence. The game ends by discussing the importance of applying step 5 to achieve ongoing improvement. Furthermore, the participants discuss whether implementing the TOC in a real ED or any other healthcare environment would bring benefits.

### 2.1. Processing Policy

During the first shift, the four resources at the top of Figure 1a (SUTURE ROOM, IMAGING EXAMS, LAB EXAMS, and MEDICATION) adopt a policy based on common practice in healthcare and other environments of worker efficiency. As the facilitator explains, a few years ago, the manager decided to improve the local resource efficiency by decreasing setup times of these resources and processing patients in batches. Thus, players can only roll the dice when three or more patients are waiting at these resources. However, every round one of these resources does not move a patient, this resource receives a delayed care token. When a resource has two tokens and fewer than three patients, it cannot roll its die, and one of these patients leaves the ED without treatment—a left-without-treatment (LWOT) patient. Sivey [26] describes this situation as follows: the longer the waiting time, the higher the probability that more low-urgency patients leave without being treated (see Figure 1b). You can remove all the tokens from a resource once a die can be rolled (the resource has three or more patients waiting) or when there are no patients waiting. After playing the first shift, the players can decide whether to keep this batching policy or not.

### 2.2. How to Measure Performance

The players will assess their performance during the game, particularly after each shift, based on three primary measurements:

- Discharges—the number of discharged patients per shift (throughput).
- Patients in treatment—the current number of patients in the ED.
- Left-without-treatment (LWOT) patients—the number of patients that left the ED without completing their treatment. If a player wants to be a good healthcare manager, no patient should leave without treatment.

**Figure 2:**
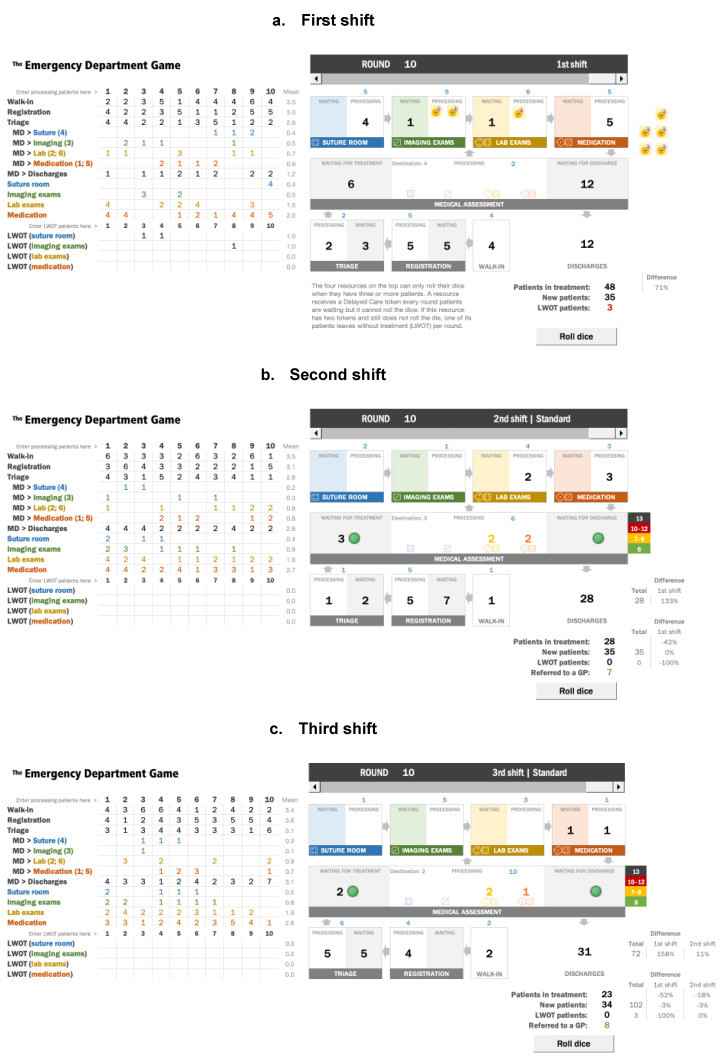
Game score sheets, including all the three shifts in the standard mode. (**a**) Shift 1 is equivalent to Strategy I, (**b**) Shift 2 is equivalent to Strategy IV, and (**c**) Shift 3 is equivalent to Strategy V (see the description of each strategy in Section 2.3).

To support measuring performance, game score sheets—a set of spreadsheets designed for this purpose—can be used. The players should record the number of walk-ins, processed patients, and the number of patients that left the ED (LWOT and discharged patients) during each round. The link to the spreadsheet is in the Supplementary Materials. See examples in Figure 2.

### 2.3. Statistical Analysis

A simulation model of the ED Game components and rules was constructed to assess and validate its gameplay dynamics and results (particularly the changes made in the game between the shifts). Next, we used a Monte Carlo (MC) simulation of 10,000 runs (complete shifts) to compare shifts 1, 2, and 3 and alternative strategies (described below). This simulation evaluated the probability of undesired results (e.g., increasing crowding or LWOT patients) and considered the possible use of other parameters (e.g., adding a resource before making better use of the existing ones) to deepen the understanding of the concepts in the game.

The model considered the standard mode, where each shift starts with a fixed number of patients (*n* = 28). The game starts with 28 patients because this is the mean result (3.5) of a six-sided die roll multiplied by those eight areas that process patients on the ED board. Therefore, this model provides reproducible and comparable results between runs and strategies.

The simulation modeled six different strategies for the MEDICAL ASSESSMENT die (where there is a decision to be made):

I. Traditional + random: The simulation considered the traditional management (shift 1) and assigned a randomly distributed value obtained in the die roll in each round to each MEDICAL ASSESSMENT task (processes the patients waiting for discharge and the patients waiting for treatment).
II. Traditional + 50/50: The simulation considered the traditional management (shift 1) and assigned 50% of the points (the value obtained in a die roll) to each side. In case of rolling an uneven number, the patients waiting for treatment had priority. Unbalanced assignment of points also occurred when there were fewer patients in the queue to meet the 50% value on any side.
III. Traditional + discharge priority: Considered the traditional management (shift 1), the simulation assigned the maximum number of points to process patients waiting for discharge and used the rest to process those waiting for treatment.
IV. TOC + Buffer Management: This is the strategy utilized for shift 2 after the changes made by means of the TOC analysis using the 5FS and Buffer Management. The level of buffer penetration in each of the queues (prioritizing waiting for treatment) indicates how to distribute the number of points. The shift 1 debriefing section provides more details on this strategy.
V. TOC + Buffer Management: This is the strategy used during shift 3 and it is similar to the previous one (IV), except for the extra resource (die) allocated in MEDICAL ASSESSMENT resulting from step 4 “Elevate”. The shift 2 debriefing section provides more details on this strategy.
VI. Traditional + Elevating first: Since the usual solution to ED crowding is to pay for more resources, we investigated the results of adding more resources (an extra die) before making a better use of the existing ones.

The simulation also supported the following parameters to select:

- A minimum batch: To determine the minimum number of patients needed to activate a resource in the top row (e.g., IMAGING EXAMS, MEDICATION).
- Refer to GP: To determine whether TRIAGE refers patients to external GPs, effectively discharging them from the ED, or not.
- MEDICAL ASSESSMENT dice: To select one of the three types of dice used in this area: a standard six-sided die (D6) at shift 1, a 4–6 die at shift 2 (as explained in the debriefing of shift 1), two six-sided dice (2 × D6) at shift 3.

For each strategy (considering 10,000 runs), we measured the mean (μ), median, variance, standard deviation (σ), and minimum and maximum values considering a 95% confidence level of the number of patients discharged (total and per round), including regular discharge and referrals to external GPs. Moreover, we measured the means of the following numbers:

- Patients in treatment in the ED (which can be considered as a work in progress (WIP)).
- LWOT patients.
- Output lead time (including LWOT and discharged patients).
- Length of stay (LOS)—the total LOS (the number of rounds it takes for a patient to leave the ED, by discharge or without treatment) and the LOS to the discharge (the number of rounds it takes for a patient to be discharged and/or referred to an external GP).

### 2.4. Pilot Implementation

At the end of 2019, one author facilitated the pilot version of this ED game with success in a limited-size workshop. The audience consisted of physicians, C-level managers, a consultant (from a major healthcare organization), a pharmacist, and IT professionals—all the participants worked in the healthcare market, mostly in major hospitals in Portugal. The objective of applying the pilot during the workshop was to assess and validate the cause-and-effect relationships, the gameplay, and the players’ experience. The workshop featured 12 participants, and nine of them played the game. The author who facilitated the gameplay observed the participants during the gameplay assessing their involvement, understanding, and capacity to provide solutions. After the workshop, some participants answered a brief survey.

This article details the experience of running this game in this workshop (according to the workshop survey’s results and the players’ comments during and after the game) and other moments of individual tests. After playing the game, the players were able to recognize that the problems presented in the ED Game also existed or were representative of similar problems in their healthcare environments, and these problems were also caused by traditional management practices. Furthermore, they were able to develop solutions both for the game and for their environments.

## 3. Results (Playing the Game)

In this section, we describe the instance of the ED Game within the context of the workshop. Each subsection describes a complete shift in the game. All the subsections contain a narrative description of the gameplay, followed by the statistical results from the Monte Carlo (MC) simulation, and a debriefing session that describes the concepts presented to the players, including a summary of their discussion to solve the game. The debriefing session introduces the TOC for those readers that are not knowledgeable of this management philosophy. However, it is beyond the scope of this article to provide exhaustive training on the TOC. Those who are knowledgeable of the TOC can use this article as a guide on how to apply this game to a healthcare audience. Those who want to learn more specifics about the 5FS and Buffer Management and the basics of the TOC should visit the Theory of Constraints International Certification Organization website [27].

### 3.1. Shift 1—Starting the Game

#### 3.1.1. Play

After playing for a few rounds, patients start to accumulate in MEDICAL ASSESSMENT and the player responsible for this resource usually complains about the players responsible for TRIAGE and WALK-IN. As the situation deteriorates, other players start complaining to the one responsible for WALK-IN every time this player rolls a 5 or 6, sometimes even a 4.

When MEDICAL ASSESSMENT is overcrowded, the players start discussing which type of patients MEDICAL ASSESSMENT should process. Some argue that they should reduce ED overcrowding by focusing on the processing of the patients waiting for discharge. However, when MEDICAL ASSESSMENT processes the patients waiting for discharge, the patients waiting for treatment accumulate and the specialized resources at the top row may be idle. At this stage, other players argue that they need to process the patients waiting for treatment. This situation worsens when patients start leaving without treatment. Now, the players start questioning the patient-“batching” policy. Figure 2a illustrates typical results for the end of the first shift.

#### 3.1.2. Simulation results

The MC simulation showed that we could expect to discharge a mean of 15.6 patients (σ = 3.11) when prioritizing discharge. The LOS to the discharge (rounds from WALK-IN to DISCHARGE) was 19.4 rounds; the mean of LWOT patients was 11.2. Although prioritizing discharge was the best strategy, its results are still statistically similar to the other strategies. Table 1 summarizes the results of the MC simulation.

#### 3.1.3. Debriefing

At the end of the first shift, the facilitator stimulates the players’ discussion by questioning them about systems thinking, constraint identification, and flow concepts. This Socratic approach ultimately leads the participants to recognize the importance of the abovementioned concepts.

The first step is to question the existence of a real need to change, “Why change?”. It is a consensus that this emergency department needs to change. What are the system problems—undesirable effects, UDEs in the TOC terminology? Let us list some of them:

- Overcrowding (high bed occupancy rate).
- Delayed assessment or treatment (and consequentially worse outcomes in the real environment).
- High workload (which may lead to a shorter time to investigate patients’ conditions in the real environment).
- High stress for staff (which is related to compromised quality of care in the real environment) and patients.
- Low discharge rate.
- Patients leaving without treatment.
- Poor performance, low efficiency, and high cost of care/treatment.
- Ambulance diversions to other hospitals’ EDs.

Do these UDEs exist in reality? According to the physicians who played this game and many published articles [2,3,26,28,29], yes. However, like a disease, these are just symptoms of the core problem of using the traditional management system. Let us analyze this system using the TOC methodology. First, let us define our system’s goal. This is not a difficult task but it is often either assumed or overlooked. As the players could see, providing excellent care is not enough; the ED must provide timely care and, in real life, it must operate at a profit or within budget (depending on the purpose of the organization). Thus, players agree with the following goal: “To provide excellent timely patient health care at a profit (or within budget).”

When playing the game, it seems the ED has many bottlenecks causing patient flow disruption, just like in real life. One may argue that the most significant cause is the die and its uncertainty (in other words, statistical fluctuations). Sometimes a resource needs a processing capacity of 5 or 6, but the die rolls 1 or 2. Conversely, when a resource has only few patients, the die might roll 5 or 6! This demand and supply fluctuation occurs in real life, too. Sometimes patient demand is high, and other times it is low. Sometimes a physician can take longer than expected to see a patient, and other times it is faster than expected (i.e., effective capacity is high/low, respectively). In addition, treatment time in a given process varies significantly; sometimes treatment time is low, and other times it is quite high. These wide fluctuations of dependent resources create chaos in a system unless managed properly. How to properly manage this environment is the question.

**Figure 3:**
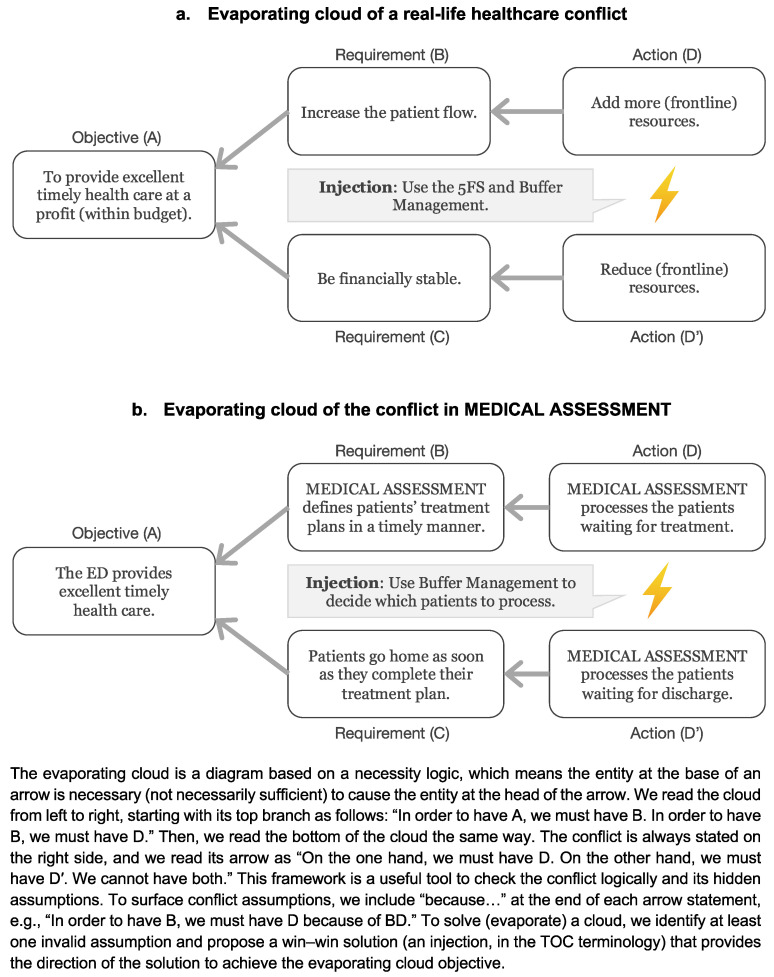
Evaporating clouds illustrating a real-life healthcare example (**a**) and the game (**b**). An explanation about the cloud is at the bottom.

Organizations encourage a local optima mindset. Every resource wants to work with 100% efficiency, but uncertainty in a prior resource seems to keep ruining the plan. Figure 3a illustrates this universal healthcare conflict [18] as an evaporating cloud—a TOC tool to precisely define a problem [22]. To achieve its goal of (A) providing excellent timely health care at a profit (within budget), a healthcare organization must (B) increase the patient flow. Statistical fluctuations delay the patient flow, and the usual solution is to (D) add more (frontline) resources. On the other hand, to achieve its goal, a healthcare organization must (C) be financially stable, which means (D’) reducing (front-line) resources.

The traditional management approach tries to find an optimal solution where both parties must compromise in the short term. However, this solution is not sustainable, and soon the conflict arises again. The TOC can successfully increase the patient flow because it makes a better use of the existing resources by applying the 5FS. Furthermore, the TOC uses Buffer Management to protect the systems’ constraints from statistical fluctuations. Hence, the TOC eliminates the conflict by achieving both requirements (B and C) without compromising any side—a sustainable solution.

One solution to “evaporate” this conflict is to use the TOC’s Five Focusing Steps (5FS) [30] to improve this system with the existing resources:

1. Identify the constraint(s).
2. Decide how to exploit the constraint(s).
3. Subordinate everything to the above decision(s).
4. Elevate the system’s constraint(s).
5. If in the previous steps a constraint has been broken, go back to step 1, but do not allow inertia to cause a system’s constraint.

The first step is to identify the system’s constraint. Every organization has a constraint, otherwise its capacity would be infinite. Since there is a constraint at any point, how can we identify it? Sometimes it may look like there are too many bottlenecks, or it looks like the bottleneck is wandering, moving from resource to resource. In most cases, scheduling or batch-sizing rules (like the patient-“batching” policy in the game) cause a wandering bottleneck. Frequently, only one constraint exists for any processing line. Once the organization starts practicing constraint management, the bottleneck usually stabilizes [31].

Identifying the bottleneck takes some work. Often, the best method is to walk on the floor observing the action and ask those employees who are familiar with the workflow throughout the production process. They are often able to point out one or two resources as the constraints. Checking the overtime records also helps to clarify this issue [31]. In healthcare environments, the constraint is usually the providers, beds, or the operating theater.

After the first shift of the game, the players can promptly say which resource is blocking them from achieving the goal—to process more goal units (in this game, patients). They correctly say MEDICAL ASSESSMENT. See Figure 2a.

In step 2, we must determine how to exploit the system’s constraint(s), that is, how to make the maximum effective use of it. Actions to better exploit the constraint include reducing idle time and eliminating (or reassigning to other resources) the tasks the constraint should not do (e.g., perform low-skill-level tasks). The constraint should perform only high-skill-level tasks, focus on the current patient, eliminate multitasking, have all the needed supplies, information, equipment, etc. to treat the current patient at hand.

In this game, MEDICAL ASSESSMENT is the constraint. As explained earlier, the constraint determines the system’s throughput. Therefore, if we want to improve the system’s throughput, we must do everything possible to make the constraint focus on performing only high-skill-level tasks. In a real-life setting, physicians should not waste time doing tasks that other qualified (and less expensive) professionals could do, like measuring blood pressure. The players should discuss actions to better exploit the constraint, such as reducing idle time and eliminating (or reassigning to other resources) the tasks a physician should not perform. In an ED, that may correspond to distinguishing high-severity patients from those with lower severity. Those low-severity patients can wait in a chair and follow a different path (e.g., a primary care track), while high-severity ones can rest on a bed and benefit from more attention from physicians.

As a result of their improvement efforts, players can expect an improvement in processing capacity at the constraint resource. To reflect this improvement, the constraint resource’s die should consider the following value changes: 1 = 4, 2 = 5, and 3 = 6. A uniform distribution of a regular die varies between 1 and 6 and has a mean of 3.5. The proposed change will improve the mean from 3.5 to 5, a 43% improvement in processing capacity. Moreover, this change will significantly reduce the uncertainty (lower standard deviation) and improve the resource’s mean processing capacity. Although it may seem to be a huge improvement without adding new resources, it is perfectly reasonable for a TOC’s implementation as reported by Mabin and Balderstone [14,32], who reported a mean reduction in lead time of 70%, and by Bacelar-Silva et al. [13], who reported a mean improvement of 61% in the percentage of the patients seen and released within the 4 h target at Accident and Emergency Departments and a mean reduction of 38% in the LOS (in a rich collection of environments, e.g., whole hospitals, specific wards, and emergency departments).

As mentioned earlier, do all patients that go to an ED need this specialized care? No, having access to a primary care provider would reduce non-urgent ED visits by 40%, as a Canadian study concluded [33]. Furthermore, some low-urgency patients leave the hospital during the waiting period without being treated when waiting times are high [26], wasting provider care capacity by not completing patient treatment. For that reason, TRIAGE first refers one patient to a general practitioner (GP) every time the die rolls 2 or more, and the remaining ones go to MEDICAL ASSESSMENT. It is important to distinguish a referral to a GP from an LWOT situation. In the former, the patient is properly assessed and referred to continue treatment in another place, while in the latter, the patient leaves the ED without clinical consent.

Another common and underestimated problem in real EDs is that professionals usually do not have a priority list to process patients when the clinical condition is no longer a differentiator. In this game, this situation occurs more often in MEDICAL ASSESSMENT. Which patients should doctors see, those waiting for treatment or those waiting to be discharged (in real life, these patients may need more treatment)? An evaporating cloud provided in Figure 3b illustrates the conflict in MEDICAL ASSESSMENT of prioritizing either treating patients or discharging patients. To address this issue, let us use Buffer Management, a TOC method to address priorities, reduce the impact of uncertainty, and provide ongoing improvement.

Buffer Management is a process of ongoing improvement and has four main functions: (1) prioritizes the tasks based on Buffer Consumption, (2) signals when to expedite the tasks at risk, (3) provides feedback, and (4) identifies the major causes of delay. The buffer is protection against the uncertainty that may take many forms (e.g., time, stock, capacity) and is strategically located to protect the system from disruption. The buffer is usually divided into three zones (green, yellow, and red), each one representing 1/3 of the total buffer. If the buffer is green, no action is required (everything flows smoothly); if the buffer is yellow, a potential problem exists (plan accordingly to address the problem); if the buffer color is red, the problem is imminent, address it (implement the plan previously made) [22].

In healthcare environments, Buffer Management can have many applications. Umble and Umble [16] described its use in EDs in the UK, where patients must be released (discharged or wait for admission) within less than 4 h after arriving. The buffer had three zones of 1 h and 20 min each. The patients who had just arrived were in the green zone, then moved to the yellow, and later to the red. The black zone grouped those patients who were still in the ED after completing the 4 h. Cox et al. [34] used examination rooms with prepared patients as a buffer to protect providers (the constraint) from idleness in a primary care setting. Bacelar [35] used chairs with prepared patients to protect the provider (the constraint) in an ophthalmology setting.

For the purpose of the game, let us consider the ideal number of patients waiting at each side of MEDICAL ASSESSMENT as six patients or less (because this is the maximum number of patients this resource can process in each round). Now, let us establish the buffer zones: green, yellow, red, and black. When MEDICAL ASSESSMENT has six or fewer patients, the buffer is green (everything operates correctly); when it has 7–9 patients, the buffer is yellow (a potential problem might occur, plan accordingly); when it has 10–12 patients, the buffer color is red (activate the plan); and when it has 13 or more patients, the buffer is black (the problem has materialized, take additional actions to get back on schedule). The priority in the game should be discharging patients, but using Buffer Management, players will notice when to dedicate more attention to processing the patients waiting for treatment.

With such policies and supporting rules, players will improve the use of the constraint resource that dictates the system’s throughput and lead time. However, it is not all we can and should do; we must implement step 3. Despite the new rules to make a better use of the constraint, the collaboration and synchronization of all other resources is necessary because they are an integral part of the system, a series of dependent events.

With that in mind, players are asked what they would change to improve the system; support a continuous flow for MEDICAL ASSESSMENT. Remember the patient-“batching” policy? This policy was initiated to make each of these specialized resources more efficient but recognize that these resources are non-constraints. This policy also has the unintended consequences of blocking the patient flow, which increases the patient time in the system and causes some patients to leave the ED without being treated. Knight [18] also describes batching policies such as batching test results in his book *Pride and Joy* based on real situations faced as a TOC healthcare consultant. Is there a real need for this batching policy? Not really. As those resources subordinated to the batching policy are non-constraints, they do not need to pursue local optima (e.g., improve worker efficiency in performing their tasks). Both constraint and non-constraint job descriptions should be rewritten. The constraint resource should focus on the task at hand, perform only high-skill-level tasks, and have all the information, supplies, and support available to perform tasks on the current patient. Non-constraints have to change their mindset from them individually being highly efficient to identifying and sequencing their individual tasks to fully support the constraint resource and improve the patient flow. Taking this into consideration, should the batching policy be removed? Yes.

Notice that up to this point, there was no need to invest money in buying new equipment or increase operational expenses by hiring new professionals (this is step 4). All the improvement actions taken focused on making a better use of the system’s existing resources to improve the constraint utilization and patient flow. Now, the team can play again.

### 3.2. Shift 2—Improving the System

#### 3.2.1. Play

The game restarts with 28 patients distributed as shift 1. After a couple of rounds, players notice a dramatic improvement in the system. They feel more confident as the patient flow stabilizes and the system can now treat and discharge more patients. In the game illustrated in Figure 2b, the throughput increased from 12 to 28 (+133%), without considering those nine patients referred to a GP, totaling 37 properly discharged patients. As a consequence, the number of patients in treatment was reduced by 20 patients (−42%) when compared to the first shift, which had the same number of new patients entering the ED. Furthermore, the second shift had no LWOT patients (a clear quality improvement).

Players also appreciate using Buffer Management to help them manage the flow. All players notice it is much easier to understand when the constraint is at risk, particularly the player in MEDICAL ASSESSMENT. When the constraint runs out of patients but the system is not empty, Buffer Management also helps players to search for and identify the major causes of disruption instead of blaming someone else. Once these causes are identified and eliminated, the patient flow is much smoother and the lead time is reduced.

*Simulation Results*

**Figure 4:**
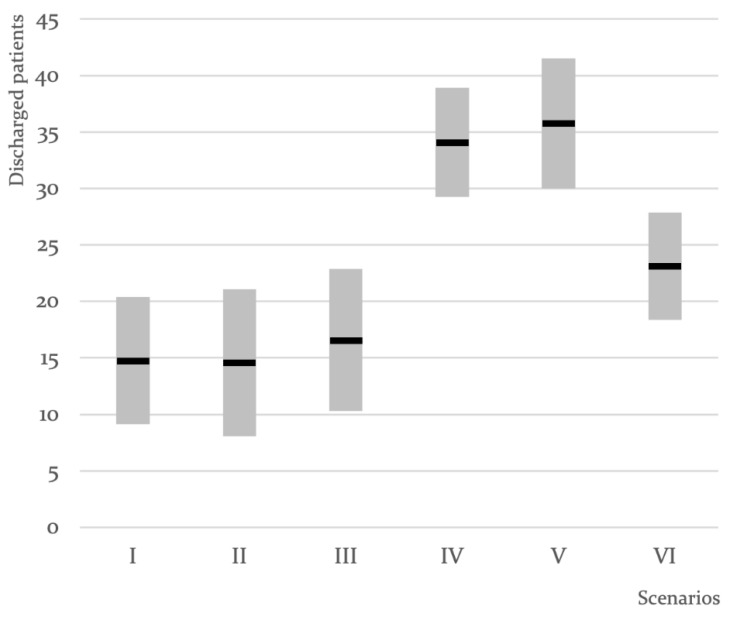
A chart illustrating the mean and the confidence interval (95% confidence level) of the number of patients discharged (total and per round), including regular discharge and referrals to external GPs, for all the different strategies. Strategies I, II, III, and VI—traditional; strategies IV and V—TOC.

The improvement after playing with the TOC approach (Strategy IV) is visible in the MC simulation results. The mean discharge was 34.1 patients (σ = 2.42), a statistically significant improvement since there is no overlap between its confidence interval and those from the traditional approach (Strategies I, II, and III) considering a 95% confidence level (see Figure 4). The lead time (LT) to discharge was reduced dramatically, from 19.4 rounds to 8.4 rounds. Since the batching policy no longer existed, there were no LWOT patients. Table 1 summarizes the results of the MC simulations.

#### 3.2.2. Debriefing

After playing the second shift, the players noticed they could increase the patient flow using the same resources. In an actual environment, that means keeping financial stability—a win–win solution. That is the aim of the TOC solutions: always try to achieve both requirements (B and C) of the evaporating cloud. However, players only applied the first three steps of the 5FS.

Now, the facilitator must ask players if they think their ED still has room for improvement. Their ED is already treating and discharging more patients than ever before. Because of that—the facilitator says—the ED has a higher budget (which may be due to many reasons, such as eliminating the need for overtime or because its funding is based on the number of patients treated) and can buy new equipment or hire another professional, which means players can allocate a second die to a resource. This is step 4, elevate the constraint. Only now players should consider investing or increasing costs, but always because there is still room for increasing throughput, e.g., the number of patients treated.

In real life, everyone using the traditional management philosophy would have a great idea to improve a particular silo, and people think it would bring great results to their silo. However, will any local improvement bring great outcomes to the organization as a whole? Organizations still have limitations to invest in terms of money, attention, and resources. With that in mind, where would someone focus the organization’s investment and improvement efforts? In 2005, a hospital decided to expand its ED, from 28 to 53 beds. Five months later, the total LOS increased and ambulance diversion episodes continued despite the expansion [36]. According to the authors, “ED expansion appears to be an insufficient solution to improve diversion without addressing other bottlenecks in the hospital.” Mumma et al. reported a similar conclusion in another study [37].

The implementation of the previous three steps usually reveals an enormous hidden capacity. However, suppose the system’s constraint is already working at its limit based on implementing the first three steps and the system still needs to increase its throughput. Therefore, this is the moment to consider an investment or an increase in operating expenses. In this case, the return on investment is often within a few months because there is a validated and high enough demand to justify the investment.

Back to the game, where would the players allocate this extra die? Recall the goal of the ED is to increase its throughput (defined as the number of patients provided excellent timely health care). What limits the ED to achieve that goal? All players agree where to focus their investment and efforts—the constraint. Now, the players can play the last shift rolling two dice for MEDICAL ASSESSMENT.

### 3.3. Shift 3—Last Attempt

#### 3.3.1. Play

Playing with two dice in MEDICAL ASSESSMENT provides extra system capacity, and the players noticed the throughput increased from 28 to 31 (+11%), see Figure 2c. However, it was not a dramatic improvement compared with the second shift when they achieved 133% improvement by using the first three steps of the 5FS. The number of new patients in the third shift was similar, decreased by one patient (−3%), and the number of patients in treatment reduced by five patients (−18%). Again, this shift had no LWOT patients, and eight patients were referred to a GP.

What happened in the game? Why was the improvement so small this time? The constraint moved.

#### 3.3.2. Simulation results

The MC simulation results demonstrate that there is a slight improvement (from Strategy IV to Strategy V) after playing with an extra die at MEDICAL ASSESSMENT. The mean discharge was 35.8 patients (σ = 2.89), which was not a statistically significant improvement since there was an overlap between its confidence interval and those from the traditional approach considering a 95% confidence level (see Figure 4). The lead time (LT) to discharge reduced slightly, from 8.4 rounds to 7.8 rounds. Since there was no batching policy, there were no LWOT patients. Table 1 summarizes the results of the MC simulation.

Furthermore, we analyzed the result of adopting the traditional solution proposed to solve the ED crowding issue: adding a new resource—step 4 (Table 1, Strategy VI). Adding an extra die at MEDICAL ASSESSMENT led to an improvement when compared to shift 1; the mean discharge increased from 15.6 patients to 22.7 patients (σ = 2.59). However, such improvement was not statistically significant (see Figure 4). On the other hand, the TOC results (strategy IV and strategy V) were still significantly better than when adding an extra die (strategy VI) since there was no overlap between its confidence interval and those from the traditional approach considering a 95% confidence level.

#### 3.3.3. Debriefing

Players notice that MEDICAL ASSESSMENT is not where patients are crowding anymore. Most likely, players will complain about TRIAGE or REGISTRATION. Either way, the 5FS is a process of ongoing improvement, and after all the efforts, the players have broken the constraint.

The lesson to be learned at this stage is the 5FS’s last step is of utmost importance: if in the previous steps a constraint has been broken, go back to step 1, but do not allow inertia to cause a system’s constraint. The application of the fifth step is a necessary condition for establishing a successful process of ongoing improvement. Inertia may misguide players and TOC practitioners to keep improving MEDICAL ASSESSMENT when it is no longer the system’s constraint. After all improvement efforts, the constraint is most likely TRIAGE or REGISTRATION.

## 4. Discussion

Statistical fluctuations and dependent events exist in all organizations at high levels. It is virtually impossible to achieve effective management practicing the traditional management philosophy. In these environments, constant firefighting becomes the norm. Telling managers and workers that the way they manage is the problem—while they try their best—results only in a communication failure. They must experience the impact of these facts of life (statistical fluctuation and dependent events) themselves to better understand the core problem.

The ED Game reproduces a more familiar environment and dynamics for healthcare professionals. By playing this game, participants can visualize the impact of their local decisions in the system as a whole in a ludic, controlled, and non-threatening environment. In addition, it provides the foundation for building simple but highly effective system solutions. We expect that the ED Game can support both practical implementations and academic applications of the TOC in health care. In this section, we discuss the theoretical and practical implications of the game.

### 4.1. Theoretical Implications

A literature review conducted by Mabin and Balderstone [32] assessed the results of the TOC’s implementations from 77 different companies. These companies had various distinctive purposes (government, not-for-profit, and for-profit) and differed in industries and sizes. The authors demonstrated that the TOC allowed organizations to increase their capacity by using the existing resources. A more recent study [13], which included 42 case studies/implementations, confirmed that the same was true for healthcare services. The authors of both studies reported rapid breakthrough results in the short term and no need for significant investment. The ED Game successfully reproduces the outcomes reported by these studies. The statistical analysis results validated the gameplay and the difference between the TOC and the traditional strategies, including the most common one—adding more resources first.

What makes the TOC different from other management approaches is the fact the TOC’s implementations focus the improvement efforts. The 5FS and Buffer Management indicate how to make a better use of the constraint that limits the whole system. Recall all the resource job descriptions were restructured. The new job descriptions must support the constraint, allowing it to perform only high-skill-level tasks. In addition, non-constraint resources must prioritize, synchronize, and execute tasks to support the increasing constraint utilization and patient flow. When any investment is necessary, it is after a thorough analysis, and the return is expected to occur in a relatively short time [13,14,32,38]. That means the TOC allows organizations to achieve a much better system performance using the existing resources. Furthermore, the TOC provides tools to continuously improve the organization because a constraint will always exist. The gameplay supports these operational characteristics and the TOC’s application throughout its three shifts. Shift 1 allows participants to apply their existing knowledge. Strategies I, II, and III are examples of such strategies. Shift 2 stimulates participants to apply the TOC’s principles and tools (Strategy IV). Shift 3 lets participants experience adding an extra resource, but only after better exploiting and subordinating the existing resources (Strategy V).

Despite the extraordinary benefits of a given new management approach, when someone tries to introduce it to solve a chronic problem, there is a natural resistance by those who have been long involved in the system chaos. After all, chances are they have already gone through many prior initiatives that failed to deliver the expected benefits despite the huge efforts of everyone. Then, it is a common thought: “If people are working as hard as they can, what would make a difference but more resources?”, which means jumping straight to step 4 (elevate the constraint). This game’s dynamics teach participants to avoid jumping straight to the usual solution of adding more resources. In addition, it supports finding solutions based on existing resources first.

### 4.2. Practical Implications

Crowding in the ED, ambulance diversions, lack of beds in hospitals, cancellation of elective surgeries... they all share a common cause. Healthcare organizations manage their resources and the patient flow based on bad/outdated management policies. Managers try to improve local efficiency everywhere (all the workers must be busy all the time), such as by balancing capacities [13]. Those who played the ED Game understood how bad/outdated policies impact the patient flow and cause undesirable outcomes. During the gameplay, the participants learned how to improve the flow, eliminating or significantly reducing the undesirable consequences. Moreover, they learned how to provide those desirable effects, such as providing timely, excellent health care by using the existing resources. If the participants of the ED Game successfully apply this recently acquired knowledge in their healthcare environments, they can achieve a significant jump in performance. That would prevent patients from the long wait for healthcare delivery and its serious consequences—emotional and clinical ones [9]. Ultimately, this improvement would contribute to avoiding illnesses aggravation, including decreased mortality.

Recall the healthcare organizations that applied the TOC described significant improvement in the short term using the existing resources, just like the experience of playing the ED Game. Umble and Umble [16] described a significant decrease in waiting times and admission times after applying the TOC in the EDs of three hospitals in the United Kingdom. Before the TOC, these hospitals could not meet the required percentage (90% or more) of the patients spending less than the 4 h limit adopted by the government. The Milton Keynes District Hospital’s average varied between 60% and 75%; the Oxfordshire Horton Hospital’s and the Oxfordshire Radcliffe Hospital’s average values varied between 50% and 60%. After the TOC, the percentage of patients seen within the 4 h limit increased to over 95% (in 5 months), 91% (in 4 months), and 95% (in 2 months), respectively. Karvonen et al. [39] reduced heart surgery queuing times of urgent patients by over 50% (from 10 days to less than 5 days) in 5 months. Heart surgery queuing times of non-urgent patients reduced by 75% (from about 80 days to about 20 days) in 4 months. Moreover, the estimated cost savings were about 0.76 million euros per year.

The ED crowding problem has negative effects on the staff as well, mostly on nurses and physicians. Consequences involve an increased level of stress, nonadherence to the best practice guidelines, increased frequency of errors, and even increased exposure to physical violence directed toward the staff [1,2,3]. Traditional approaches do a poor job at compensating for these natural occurrences, particularly with job descriptions focused on making each job position as effective as possible at their specific tasks. Instead, managers should view the actual workflow across a series of workers and develop job descriptions to increase the workflow. The players experienced this during the game when they decided how to improve MEDICAL ASSESSMENT. The facilitator must express the need to review job descriptions to support the constraint and improve the flow. This is how a system can better exploit its constraint. Applying the TOC to a real environment brings numerous benefits to all the stakeholders without overloading anyone in particular [35,40,41]. This allows healthcare professionals to perform their jobs without pressure and provide better care. Mabin et al. described the impact of a TOC’s implementation on staff [42]. Besides reporting improvement in the average wait times of 87% for chemotherapy treatments, it provided a two-thirds (67%) reduction in nursing staff overtime and eliminated the need for overtime for all the other staff (those involved reported reduced stress levels). At the same time, it maintained an insignificant level of wastage and improved patient satisfaction.

Many people see insufficient beds as their ED’s constraint, and they may be partially correct. Lack of beds (either in the ED or in specialized wards—which causes a backup in the ED) is not the root cause of ED crowding. Consequently, the solution cannot start by adding more beds everywhere. When analyzing this situation under the framework provided by the 5FS, notice that people correctly identified the constraint (step 1), but they wanted to improve the system by adding more resources, that is by going straight to step 4 (elevate the system’s constraint). Before jumping from step 1 to step 4, people should decide how to exploit the system’s constraint (step 2) and subordinate everything to the previous decisions (step 3). This is where ED’s hidden capacity lies, underneath bad/outdated traditional management policies—the root cause of ED crowding. Adding more beds without eliminating the root cause of ED crowding will demand a high investment that may not provide the expected benefits, as evidenced by the game analysis, and even compromise the financial stability. Actually, it can even make the ED’s performance worse, as reported by Han et al. [36] and Mumma et al. [37]. Considering this “if only there were more beds” mindset, the game provides an environment with infinite bed capacity. Obviously, the crowding persists until the players apply the 5FS and recognize that the root cause of ED problems is the existence of bad/outdated management policies. Sierraalta’s [20] implementation at a hospital in Venezuela achieved a reduction of 21% in the average LOS in the ED within a month (a week later, it decreased by additional 8%, achieving 29%), the average number of available beds practically doubled, the overall service time reduced by up to 80%, and all that using the same resources and the same staff.

Playing a game can be a powerful way to overcome the resistance to change and introduce new knowledge, especially when the players are familiar with the game environment and challenge [43,44]. The Beer Game designed by Jay Wright Forrester in 1960 is still relevant today and is part of management courses at the MIT Sloan School of Management [45] and elsewhere. Another example comes from the use of board games as an engaging approach to promoting health and medical outcomes in academic, patient/hospital, and community settings. According to Gauthier et al. [43], board games can significantly improve knowledge attainment compared to other nongame conditions. The authors report that board games are primarily valuable to impact the behavior of unknowledgeable audiences or when it involves communicating complex concepts related to health. In addition, board games can effectively increase knowledge and change behavior, influencing health conditions as a consequence [43]. This game may contribute to the application and learning of methodologies other than the TOC. Lean practitioners could use the ED Game to assess and compare the lean approach with the TOC or other approaches.

In all the consultant-led TOC’s implementations, consultants use Socratic facilitation of games to address the question of how to create the change. They use interactive graphic simulation models of lines, job shops, etc. to have workshop participants identify for themselves the problems of the traditional management practice and the benefits created by using the TOC processes of ongoing improvement such as the Five Focusing Steps and Buffer Management. The result is the buy-in of participants into challenging traditional methods and taking ownership of the methods they create.

### 4.3. Overall Contribution

In this article, we described the use of this game with healthcare professionals (physicians, nurses, hospital department managers, etc.). The ED Game was designed to approximate the reality of an ED environment, not to mirror it. Some of the complexities of the ED and other healthcare environments were introduced to reflect real-world situations. The game’s focus is not on how perfectly the healthcare environment is illustrated. Rather, it was designed to conveniently illustrate the problems caused by the traditional management practices and the benefits of applying the theory of constraints approach to any healthcare environment, including the ED. The game facilitator is oriented to use the structured sequence of questions provided in this document. This Socratic approach is a fundamental mechanism to retain greater attention and enhance learning. Along with the questions, the facilitator provides players with the methods and tools to analyze the context, surface the underlying assumptions, and find solutions themselves. Following this sequence, players effectively increase their knowledge, develop new paradigms, and are rewarded with a greater ownership of an effective solution.

Given the COVID-19 pandemic, testing the ED Game in a meaningful sample of ED and other healthcare personnel playing the game was impossible. Moreover, even if we addressed the limitation of getting people together, ED (and all healthcare) professionals were overloaded with work. Therefore, they would be unwilling to participate in such a study. It would require a huge scheduling effort to put enough small groups together to meet the statistical significance criterion. Future research should include testing the ED Game on a meaningful sample of healthcare personnel. It would provide greater evidence of its usefulness and learning achieved by healthcare practitioners. We expect to continue this research when the COVID-19 pandemic subsides and a return to normalcy occurs.

## 5. Conclusions

While most TOC solutions are simple, with many viewing them as purely common sense, they represent paradigm shifts in managing complex, uncertain, and silo environments. Nevertheless, the TOC has successfully improved the care delivery provided by EDs and many other healthcare settings, and this game mimics the results seen in practice.

The Emergency Department Game (EDG) is interactive, fun to play, and was designed considering the need to use a familiar environment and dynamics for healthcare professionals. Our statistical analysis assessed six strategies based on the traditional and TOC approaches and validated the game results according to the existing literature outcomes.

The game uses a Socratic approach to enhance the importance of seamless patient flow and constraint utilization. It demonstrates the impact of statistical fluctuations and dependent processes in a complex healthcare environment and how bad/outdated (traditional) management policies block patient treatment and flow. Furthermore, the game introduces the basic concepts and tools of the TOC and stimulates players to provide insights toward the potential problems and solutions in their healthcare environments. The knowledge acquired playing the game would support significant improvement in health care delivery in the short term with the existing resources and impact long-term behaviors and decisions. Ultimately, that would represent a notable improvement in the quantity, quality, and timeliness of care delivery without increasing the costs.

## Figures and Tables

**Table 1:** Monte Carlo (MC) simulation results for the six strategies with their respective statistical analysis after 10,000 runs. Strategies I, II, and III represent variations of the traditional management philosophy with different priorities utilized for MEDICAL ASSESSMENT. Strategy IV represents the TOC approach of applying the first three steps of the 5FS and Buffer Management. Strategy V is similar to Strategy IV but includes step 4 “Elevate”. Strategy VI represents the traditional management approach after adding a new resource (die) at MEDICAL ASSESSMENT to improve the ED performance (as in Strategy V).

Strategy	Shift	Type	Assessment Strategy	Discharges (Mean)	Std Dev	%Std Dev	95% Percentile (Min)	95% Percentile (Max)	WIP (Mean)	LWOT (Mean)	Total LOS (Mean)	Discharge LOS (Mean)
I	1	Trad	Random	14.5	2.74	19%	9.0	20.0	31.2	11.3	12.6	22.5
II	1	Trad	50%	14.1	3.15	22%	7.8	20.4	31.3	10.2	13.4	23.6
III	1	Trad	Discharge	15.6	3.11	20%	9.3	21.8	28.9	11.2	11.1	19.4
IV	2	TOC	BM	34.1	2.42	7%	29.3	38.9	28.5	0.0	8.4	8.4
V	3	TOC	BM	35.8	2.89	8%	30.0	41.5	27.6	0.0	7.8	7.8
VI	1	Trad	50%	22.7	2.59	11%	17.5	27.8	27.3	9.3	8.7	12.2

## Data Availability

Available at gustavobacelar@med.up.pt upon request.

## Supplementary Materials

The following are available online at https://www.mdpi.com/article/10.3390/ijerph181910083/s1, File S1: A cooperative game designed to teach how to solve emergency department crowding by using existing resources more effectively.
