# Supplementary material for: Identifying and Addressing the Underlying Core Problems in Healthcare Environments: An Illustration Using an Emergency Department Game
Source: Int J Environ Res Public Health. 2021 Sep 25;18(19):10083. doi: 10.3390/ijerph181910083 (PMC8507676; doi:10.3390/ijerph181910083)

Supplementary Material

A cooperative game designed to teach how to solve emergency department crowding by using existing resources more effectively

Overview

In this ED Game, the players must deliver care to emergency patients (with specific treatment plans) by making them flow accordingly through 7 resources till the end of the ED episode, where patients go to the DISCHARGE area. Once MEDICAL ASSESSMENT processes patients for the first time (those waiting in the gray area on the left side), they follow through different treatment plans (paths) starting from (a) SUTURE ROOM, (b) IMAGING EXAMS, (c) LAB EXAMS, and (d) MEDICATION. Participants should play the game during 3 shifts, with 10 rounds each shift. A round represents 48 minutes in time; 10 rounds represent an 8-hour shift. Figure 1a shows the board elements prepped for the first round.

In the first shift, participants are free to play according to their prior knowledge. Next, the facilitator introduces the 5 Focusing Steps (5FS) and Buffer Management, and let participants discuss and apply the first 3 steps (how to identify and exploit the constraint and adjust the system accordingly) in the second shift. In the third shift, participants discuss and apply step 4 (elevate the constraint). The game ends by discussing the importance of applying step 5 to achieve ongoing improvement (and whether implementing TOC in a real ED environment to identify, sequence, and prioritize the new tasks, rules, and policies would bring benefits).

The round starts by rolling a die to define patient demand in WALK-IN (the number of patients entering the ED). Then, the entering patients go through REGISTRATION, TRIAGE, and MEDICAL ASSESSMENT (gray area on the left side). From this point, patients can have different treatment plans (paths), options are (a) SUTURE ROOM, (b) IMAGING EXAMS, (c) LAB EXAMS, and (d) MEDICATION. After going through their treatment plans, all patients come back to MEDICAL ASSESSMENT (gray area on the right side). A patient will be successfully treated after the last medical assessment when the pawn goes to DISCHARGE. The game illustrates the destination after ED as discharge because this is the most frequent destination after an emergency department visit, almost 90% of the time in the United States, in 2017 [17]. The round ends after rolling the dice and processing patients on every resource that has patients to process.

Game components

To play this game, you will need:

- At least 2 dice;
- 28 blue pawns (patients within the emergency department at the beginning of the game);
- 40 red pawns (new patients introduced to the ED during the game);
- 8 delayed care tokens;
- 2 buffer penetration boards;
- 1-sheet or 10-sheet EDG board – for a large group, use the 10-sheet board where each sheet represents a process – except for medical assessment, which has three sheets;
- Game score sheets.

At the end of this supplementary file, you can find both EDG board versions, delayed care tokens, and buffer penetration boards to print and play. You can download the game score sheet (an Excel file) using this link:.

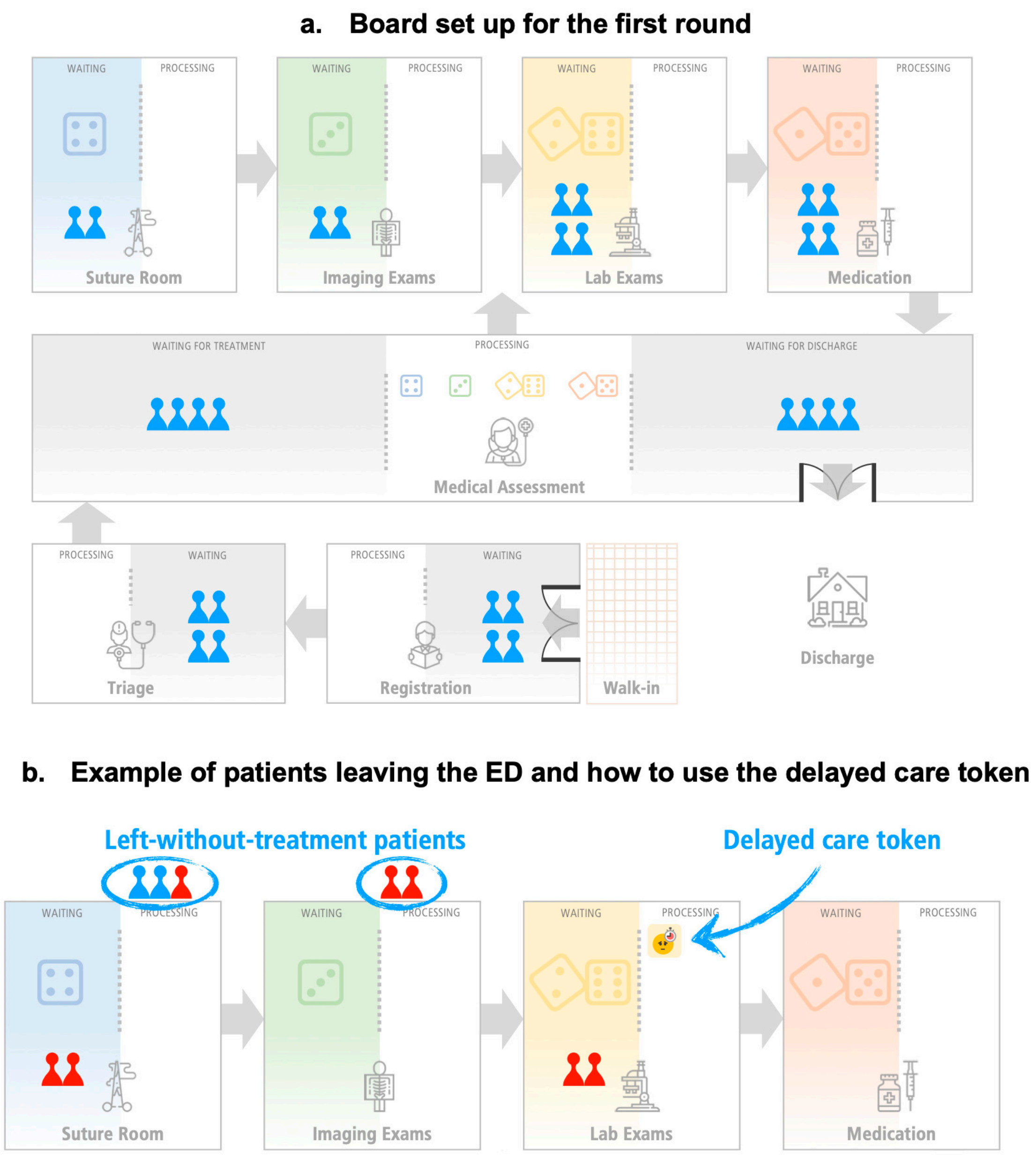

Figure S1. (a) Example of how to set up the board to play. (b) Implications of the patient "batching" policy; notice some left-without-treatment patients (LWOT) on the top of SUTURE ROOM and IMAGING EXAMS, and LAB EXAMS is at risk of receiving its second delayed care token.

Setting up the board

First, place the EDG board or assemble the 10-sheet EDG board on a table and distribute the 28 blue pawns (existing patients) placing 4 pawns on each WAITING area, except for SUTURE ROOM and IMAGING EXAMS, which receive 2 patients each. MEDICAL ASSESSMENT receives 4 on the left-handed WAITING area (colored area dedicated to patients waiting for treatment) and 4 on the right-handed WAITING area (patients waiting for discharge). See Figure 1b.

Rules

1-9 players can play this game per board. In the single-player version, that player performs all tasks. In multi-player versions, MEDICAL ASSESSMENT and the "rules enforcer/scorer" should be attributed to one player each. The other players can be responsible for a different number of the remaining resources.

Processing patients

A die roll determines the number of patients processed by a resource on each round, so the number of patients that can be seen/processed each round varies considerably. This variability of results represents the statistical fluctuation that happens in real life – where providers spend variable time to see a patient according to the clinical needs, and unexpected interruptions during a consult (e.g., interactions with support staff, phone calls, paperwork missing).

A player can only process patients available in the WAITING area (colored area). When the number on the die is higher than the number of patients available, the player is not allowed to move patients from other areas or accumulate a patient move to the next round. Processing patients stay in the PROCESSING area (white area) and will move to the next resource at the end of the round. This process repeats each round.

The player responsible for MEDICAL ASSESSMENT may have to decide to process patients waiting for treatment or those who have already been through treatment and are just waiting for discharge, see Figure 1a. The player can choose to move patients according to what s/he thinks is best (e.g., move all from one area and none from the other, move half from each area).

After choosing where to allocate the MEDICAL ASSESSMENT processing capacity, the player responsible for this resource must roll a die to define the treatment plan of those patients s/he allocated to be treated. It must happen in the sequence, not after all the other processes have moved their pieces. The number on the die will dictate where each patient will start treatment (e.g., a die roll of 1 or 5 means the patient will be allocated to MEDICATION, while a die roll of 4 means the patient will be allocated to SUTURE etc.). Allocate patients accordingly on the PROCESSING area, where they will wait until the end of the round, which prevents them from mixing these patients with those already waiting in their respective destinations see Figure 1a.

The players can roll all at once or in turn, it depends on how many dice they have. However, it is funnier to play in turns, following the ED flow. That way, they can see each other results and interact more. It is not a problem for MEDICAL ASSESSMENT to play all dice at once, patients moving to treatment will wait in their respective queue in the middle of this area. There is no risk of a patient be mistakenly available, because patients only move to another resource at the end of the round.

Processing policy

During the first shift, the four resources on the top of Figure 1a (SUTURE ROOM, IMAGING EXAMS, LAB EXAMS, and MEDICATION) have a specific policy. As the facilitator explains, a few years ago, the manager decided to improve local resource efficiency by decreasing setup times of these resources and processing patients in batches (e.g., a batch of pediatric patients waiting for a setup of the X-ray machine). Thus, players can only roll the dice when 3 or more patients are waiting on these processes. However, every round one of these resources has a patient that does not move, this resource receives a delayed care token (empty resources do not receive it).

Every round that a resource that accumulated 2 delayed care tokens does not move a patient, one of these patients leaves the ED without treatment. You can remove all tokens of a resource once you can roll the die (3 patients waiting) or all patients left it. Figure 1b illustrates some left-without-treatment patients (LWOT) on the top of SUTURE ROOM and IMAGING EXAMS, and LAB EXAMS is at risk of receiving its second delayed care token. Players can decide whether to keep this policy after playing the first shift.

Moving patients

Each process has 2 different areas. The colored area, WAITING, accommodates patients processed by a previous resource. Patients being processed during a round should move to the white area, PROCESSING. Before starting to play another round, the players

must move patients from PROCESSING areas to the next resource's WAITING areas. Discharged patients do not stay in a PROCESSING area, they go straight from WAITING FOR DISCHARGE to the DISCHARGE area.

How to measure performance

Players will assess their performance based on 3 primary measurements:

- **Discharges** – the number of treated patients per shift, it's the system's throughput rate.
- **Patients in treatment** represent the current number of patients in the ED.
- **LWOT patients** – the number of patients that left the ED without completing their treatment. If you want to be a good healthcare manager, no patient should leave without treatment.

You can play this game in 2 different modes, continuous or standard. The continuous mode considers the results and patients in treatment at the end of the previous shift as the starting point for the next shift. Starting a new shift with patients in treatment from the last shift is a natural feature, increases the pressure for improvement, and allows the players to observe the outcomes of their actions in a challenging environment.

In the standard mode, every shift starts with 28 patients in treatment distributed as previously described in the "Setting up the board" section. In this mode, the players can use other measurements to compare their team performance, such as **lead time to discharge** (number of rounds for a new patient to exit the ED) and **lead time to treatment** (number of rounds for a new patient to be processed on MEDICAL ASSESSMENT).

To support measuring your performance, you can use the game score sheets – a set of spreadsheets designed for this purpose. Players should record the number of walk-ins, processing patients, and the number of patients that leave the ED without treatment (LWOT) and discharged patients on each round. See examples in Figure *2*.

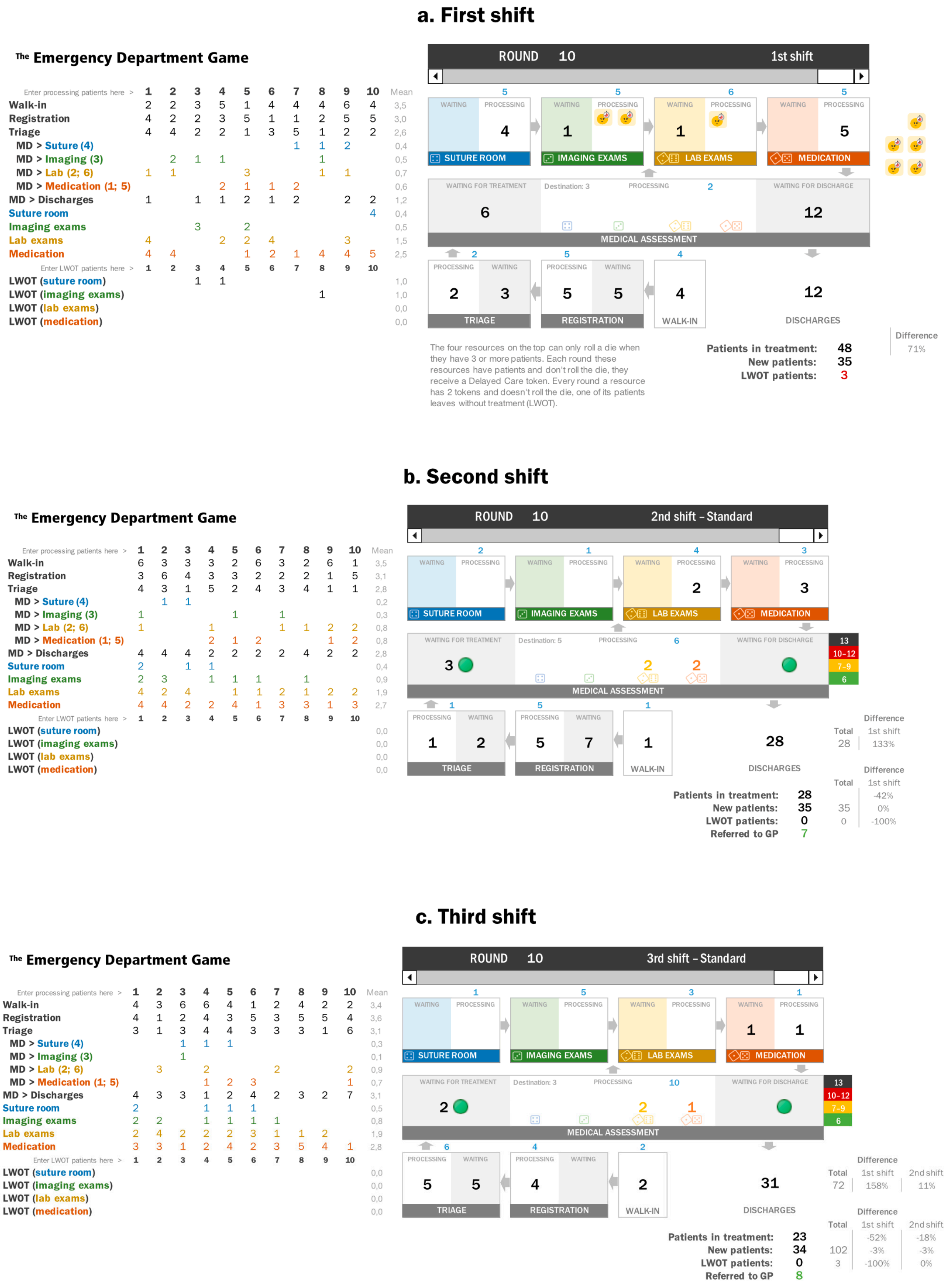

Figure S2. Game score sheets of a play, including all three shifts in the standard mode. Shift 1 is equivalent to Strategy I (traditional management + random), Shift 2 is equivalent to Strategy IV (TOC + BM), and Shift 3 is equivalent to Strategy V (TOC + BM + elevating capacity at the constraint). See the article's Table 1.

The Emergency Department Game

A Theory of Constraints educational tool designed by

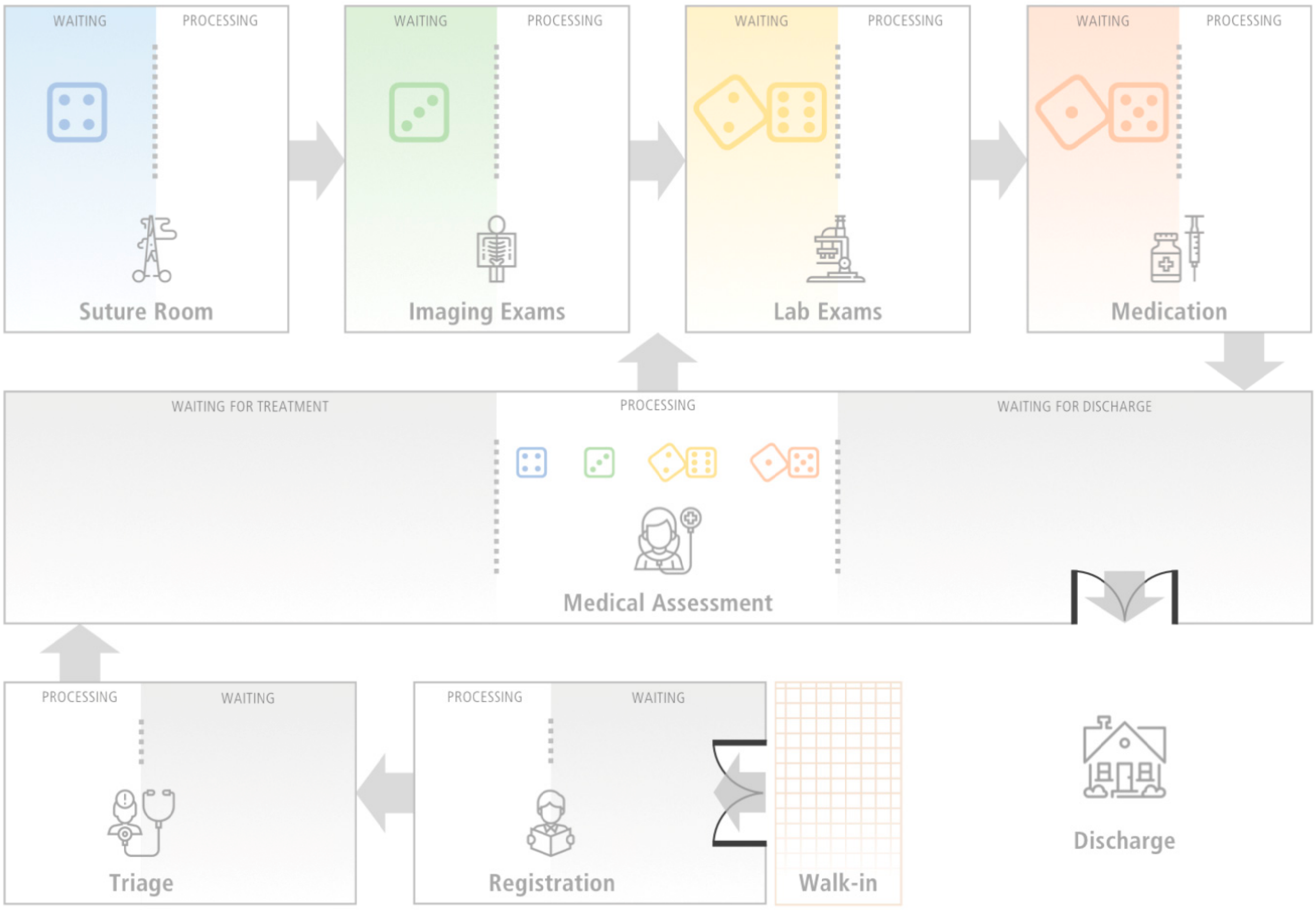

Supplement: Supplementary file 1 [file ijerph-18-10083-s001.zip › ijerph-1360746-supplementary.pdf]
